# Peripheral arterial tonometry versus polysomnography in suspected obstructive sleep apnoea

**DOI:** 10.1186/s40001-023-01164-w

**Published:** 2023-07-22

**Authors:** Jonathan Röcken, Desiree M. Schumann, Matthias J. Herrmann, Simon Veitz, Léo Franchetti, Leticia Grize, Werner Strobel, Kathleen Jahn, Michael Tamm, Daiana Stolz

**Affiliations:** 1 Clinic of Respiratory Medicine and Pulmonary Cell Research, University Hospital of Basel, Basel, Switzerland; 2 Department of Clinical Research, University Hospital Basel and University of Basel, Basel, Switzerland; 3 Department of Pneumology, Medical Center - University of Freiburg, Faculty of Medicine – University of Freiburg, Freiburg, Germany

**Keywords:** Home sleep apnoea testing, HSAT, Sleep apnoea, OSA, OSAS, Polygraphy, WatchPAT

## Abstract

**Background:**

Polysomnography (PSG) is the gold standard for the diagnosis of obstructive sleep apnoea (OSA). Home sleep apnoea testing with peripheral arterial tonometry (PAT) is a recommended diagnostic alternative for patients with an increased risk for OSA.

In a large clinical cohort, we investigated concordance and predictors for discordance in diagnosing OSA using PAT and PSG, and three-year cardiovascular risk in patients with discordant OSA diagnosis.

**Methods:**

Retrospective monocentric cohort study. Patients with a PAT AHI ≥ 5/h followed by an in-hospital PSG within three months were included. All patients with a PAT AHI ≥ 5/h but a PSG AHI < 5/h were classified as discordant. Patients with PAT and PSG AHI ≥ 5/h were classified as concordant. To ascertain cardiovascular risk, major adverse cardiovascular events (MACE) were analyzed in discordant patients and sex, age, body mass index (BMI) and cardiovascular disease-matched concordant patients over a follow-up time of 3.1 ± 0.06 years.

**Results:**

A total of 940 patients, 66% male with an average age of 55 ± 0.4 years and BMI of 31 ± 0.2 kg/m^2^ were included. Agreement in OSA diagnosis was observed in 80% of patients (55% in mild and 86% in moderate and severe OSA). Factors significantly associated with a discordant diagnosis were female sex, younger age and lower BMI, but not comorbidities. There was no significant difference in MACE (*p* = 0.920) between discordant patients (*n* = 155) and matched concordant patients (*n* = 274) with or without therapy.

**Conclusions:**

Concordance between PAT and PSG diagnosis of sleep apnoea is good, particularly in moderate and severe OSA. Predictors for discordant results between PAT and PSG were age, sex and BMI. MACE risk is similar in those with OSA diagnosed by PAT or PSG.

**Supplementary Information:**

The online version contains supplementary material available at 10.1186/s40001-023-01164-w.

## Background

Obstructive sleep apnoea (OSA) is an important but frequently undiagnosed source of morbidity and mortality [1, 2]. It is characterized by repetitive episodes of partial (hypopnoea) or complete upper airway closure (apnoea) during sleep. Common symptoms of OSA are excessive daytime sleepiness [3], snoring, nocturia [4], nocturnal awakening, and morning headaches [5]. However, many patients are asymptomatic [6]. Reported long-term consequences of untreated OSA include increased risk for hypertension [7], heart failure [8], coronary heart disease [8], atrial fibrillation [9], stroke [10], depression and traffic accidents [11, 12]. For OSA positive airway pressure (PAP) is generally the first-line treatment that reduces daytime sleepiness [13], blood pressure [1, 14, 15] and depression symptoms [16].

In-laboratory overnight polysomnography (PSG) is generally referred to as the gold standard for the diagnosis of sleep apnoea but availability is limited due to both its complexity and cost [17]. The American Academy of Sleep Medicine (AASM), therefore, also recommends the use of home sleep apnoea testing (HSAT) devices for patients with increased risk of OSA [17]. Peripheral arterial tonometry (PAT) is an approved HSAT device that has several advantages compared to in-laboratory PSG: it is home-based, relatively cheap and data are analysed expeditiously as no manual scoring is required. Apnoea and hypopnoea lead to sympathetic nervous system activation and peripheral arterial vasoconstriction. PAT detects transient vasoconstriction in the finger which can be used as a surrogate marker for the identification and scoring of sleep apnoea [18]. The results of the existing studies and their interpretation are divergent. On one hand, a good correlation between PAT AHI and PSG AHI is shown [19–22], on the other hand, misclassification of severity and presence of OSA by PAT was described [23, 24]. In addition, severity of OSA, age and certain comorbid conditions such as arterial stiffness, may affect the diagnostic accuracy of PAT [25, 26].

We hypothesized that PAT and PSG identify the same patients as having OSA. In this study we therefore assessed the accuracy of positive PAT (AHI ≥ 5/h) compared to PSG in a large clinical cohort. We also aimed to determine predictors of discordant diagnosis of sleep apnoea between PAT and PSG. Finally, using sex-, age-, body mass index- and cardiovascular disease-matched patients, we analysed three-year cardiovascular risk in discordant patients.

## Methods

This is a retrospective, monocentric study performed at the Clinic of Respiratory Medicine and Pulmonary Cell Research at the University Hospital of Basel, Switzerland. The Study was approved by the Ethics Committee northwest/central Switzerland (EKNZ 2018-01789) and was carried out according to the Declaration of Helsinki and Good Clinical Practice guidelines. Only patients who provided written informed consent for data analysis were included in the study.

**Fig. 1:**
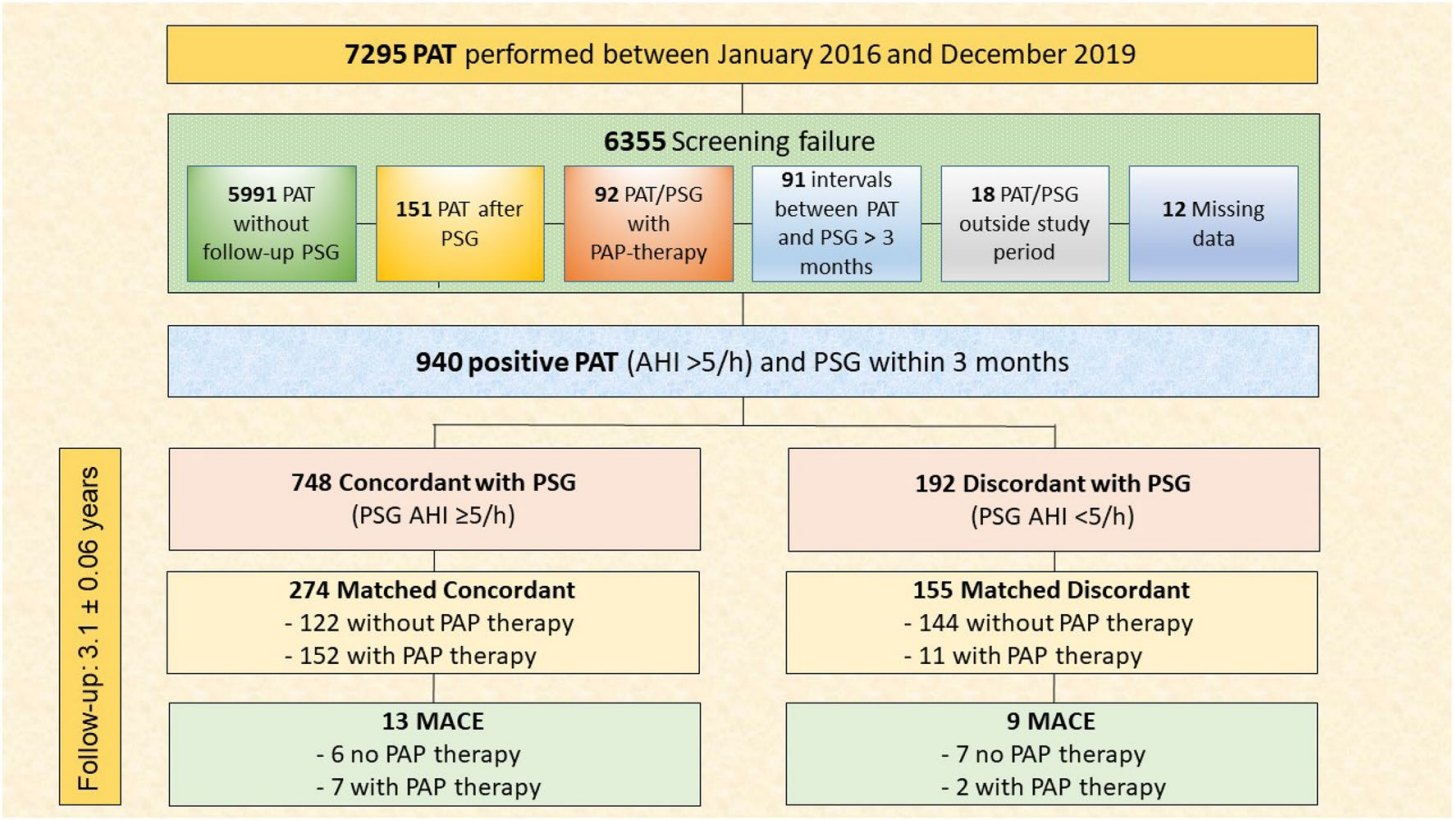
Schematic representation of patient inclusion in the study. *MACE* major adverse cardiovascular events, *PAP* positive airway pressure, *PAT* peripheral arterial tonometry, *PSG* polysomnography

Data from patients with a PAT AHI of ≥ 5/h between January 2016 and December 2019 and a follow-up PSG within 3 months of the PAT were included in the study (Fig. 1). Collected data included patient demographics, and medical history sourced from electronic health records covering both outpatient and inpatient departments of the University Hospital of Basel. Data pertaining to major adverse cardiovascular events (MACE) were additionally sourced from the primary care physicians.

WatchPAT™200 Unified (Itamar Medical, Israel) is a four-channel device worn around the wrist with a finger PAT sensor. The WatchPAT software zzzPAT version 4.4.64.4 was applied. The threshold for apnoea-hypopnoea index (AHI) and respiratory disturbance index (RDI) was a drop in oxygen saturation of ≥ 3%. The oxygen desaturation index (ODI) threshold was automatically set at an oxygen desaturation of ≥ 4%. In contrast, an oxygen desaturation of ≥ 3% is standard for setting the ODI in PSG.

All patients underwent in-laboratory overnight PSG. PSG was performed and scored (by a trained sleep physician) according to the AASM 2012 criteria [27]. Sleep apnoea was defined as AHI ≥ 5 events/h. The severity of sleep apnoea is defined as either mild (5 ≤ AHI < 15/h), moderate (15 ≤ AHI < 30/h) or severe (AHI ≥ 30/h). An Epworth Sleepiness Scale (ESS) score of ≥ 10/24 points was considered an indicator of excessive daytime sleepiness [28, 29].

In the analysis, all patients with a positive PAT (AHI ≥ 5/h) but negative PSG (AHI < 5/h) were classified as discordant. Patients with a positive PAT (AHI ≥ 5/h) and PSG (AHI ≥ 5/h) were classified as concordant. For discordant patients 1–2 comparable controls matched for sex, age (± 3 years), BMI (4 classes: 16–18.4 kg/m^2^, 18.5–24.9 kg/m^2^, 25–29.9 kg/m^2^, ≥ 30 kg/m^2^) and cardiovascular disease (at least one of the following conditions: arterial hypertension, coronary artery disease, congestive heart failure, cerebral vascular disease) were sourced from the concordant patients. In these matched discordant and concordant groups, all available data for the follow-up period until 31 March 2021 were collected. This included use of PAP therapy (number of days the device was used, number of days the device was used for more or less than 4 h), hospitalizations, MACE and mortality. MACE were defined as fatal and non-fatal cardiovascular events leading to hospitalization, including stroke, transient ischemic attack, myocardial infarction, hypertensive emergency as well as acute decompensated heart failure.

Statistical analyses were performed with *IBM SPSS Statistics for Windows, version 25 (IBM Corp., Armonok N.Y., USA).* A *p* < 0.05 was considered significant, and all tests were two tailed. Group comparisons were performed using the Chi-squared test if the variables were categorical and using the Mann–Whitney U-test if continuous. The agreement between PAT and PSG measurements was evaluated using the Lin’s concordance [30] coefficient and a Bland–Altman diagram. When calculating correlation coefficients, the Spearman correlation was used. Associations between continuous outcomes and potential predictors were examined by means of univariate linear regression models. Predictors of discordance between PAT and PSG were determined by means of multivariable logistic regression models. Cox-regression models were used to calculate the risk of suffering from MACE.

## Results

Between January 2016 and December 2019, 7295 PAT were performed of which 940 patients were included for analysis (Fig. 1). The high number of PAT examinations (*n* = 7295) in our clinic compared to polysomnography was also due to regular PAT follow-up examinations of patients under PAP therapy.

The vast majority of the PAT measurements included was performed at home (*n* = 880/940, 94%). 60 measurements were performed in hospitalised patients, but not in the sleep laboratory. The patient population was predominantly male (66%). The average age and BMI were 55 ± 0.4 years and 31 ± 0.2 kg/m^2^, respectively (Table 1). The majority of the patients were current or ex-smokers (58%). The median time between the PAT and PSG assessment was 22 days (IQR 15–35). Daytime sleepiness was present in 48% (*n* = 362/754) of the patients as assessed with an ESS of ≥ 10 points. Various comorbidities were present in the patient population. The most prevalent being arterial hypertension (57%) followed by diabetes mellitus (17%) and depression (16%).

**Table 1:** Sociodemographic and clinical characteristics of the study participants included for analysis

Characteristics	All (*n* = 940); avg ± SEM, *n* (%)	Concordant (*n* = 748); avg ± SEM, *n* (%)	Discordant (*n* = 192); avg ± SEM, *n*(%)	*p*-value^*^
Age, years	55 ± 0.4	56 ± 0.5	51 ± 1.1	**< 0.0001**
Gender				**< 0.0001**
Male	624 (66)	518 (69)	106 (55)	
Body mass index, kg/m^2^	31 ± 0.2	32 ± 0.2	29 ± 0.4	**< 0.0001**
Epworth sleepiness scale (*n* = 754)	9.3 ± 0.2	9.3 ± 0.2	9.2 ± 0.4	0.735
Time between PAT and PSG (days)	28 ± 0.7	28 ± 0.8	29 ± 1.5	0.523
Smoking status (*n* = 862)				0.091
Current/ex- smoker	301 (47)/101 (11)	155 (22)/246 (36)	51 (30)/50 (29)	
Never smoker	360 (42)	290 (42)	70 (41)	
Pack years	27 ± 1.0	28 ± 1.1	24 ± 2.0	0.052
Comorbidities	847 (90)	675 (90)	172 (90)	0.785
Arterial hypertension	534 (57)	449 (60)	85 (44)	**< 0.0001**
Asthma	125 (13)	91 (12)	34 (18)	**0.044**
Atrial fibrillation	58 (6)	48 (6)	10 (5)	0.806
Cerebral vascular disease	65 (7)	55 (7)	10 (5)	0.296
COPD	96 (10)	74 (10)	22 (11)	0.523
Congestive heart failure	69 (7)	57 (8)	12 (6)	0.516
Coronary artery disease	111 (12)	87 (12)	24 (13)	0.739
Depression	147 (16)	108 (14)	39 (20)	**0.046**
Diabetes mellitus	160 (17)	133 (18)	27 (14)	0.221
Renal disease	125 (13)	110 (15)	25 (13)	**0.012**
Rheumatological disease	76 (8)	61 (8)	15 (8)	0.877

Comparison of patients who had discordant sleep apnoea diagnoses between PAT and PSG with patients who had concordant results between the two examinations

Values in bold indicate significant *p*-values

*PAT*  peripheral arterial tonometry, *PSG* polysomnography; *COPD *chronic obstructive pulmonary disease

^*^p-value determined using Chi-square test if variable is categorical and the Mann–Whitney *U*-test if continuous

### Prevalence of OSA according to PAT and PSG

Of the 940 patients included in the study, sleep apnoea diagnosed by PAT (AHI ≥ 5/h) was confirmed by PSG in 748 patients (80%) (Table 2). Sleep apnoea severity was classified as mild in 22%, moderate in 35% and severe in 43% of the patients when diagnosed using PAT, compared to 30%, 25% and 25% when diagnosed using PSG (Table 2). Of the 192 patients where PSG did not confirm the presence of sleep apnoea, 91 (47%), 72 (38%) and 29 (15%) had been diagnosed with mild, moderate and severe sleep apnoea, using PAT.

**Table 2:** Sleep apnoea severity in PAT and PSG

		PSG sleep apnoea severity				
		No sleep apnoea; *n* (%)	Mild sleep apnoea; *n* (%)	Moderate sleep apnoea; *n* (%)	Severe sleep apnoea; *n* (%)	Total; *n* (%)
PAT sleep apnoea severity	Mild sleep apnoea; *n* (%)	91 (45)	75 (37)	28 (14)	8 (4)	202 (22)
	Moderate sleep apnoea; *n* (%)	72 (22)	126 (38)	93 (28)	39 (12)	330 (35)
	Severe sleep apnoea; *n* (%)	29 (7)	80 (20)	110 (27)	189 (46)	408 (43)
Total; *n* (%)		192 (20)	281 (30)	231 (25)	236 (25)	940 (100)

*PAT* peripheral arterial tonometry, *PSG* Polysomnography

### Prevalence of symptomatic OSA (OSAS) according to PAT and PSG

Using an ESS ≥ 10 points as an indicator of symptomatology and an AHI ≥ 5/h, we calculated the prevalence of obstructive sleep apnoea syndrome (OSAS) in our population. It was 48.0% (*n* = 362/754) based on PAT and 38.6% (*n* = 291/754) based on PSG (Additional file 1: Table S1). The average ESS was 9.3 ± 0.2 and did not correlate with PAT AHI (Spearman Rho correlation coefficient = − 0.046, *p* = 0.202) or PSG AHI (Spearman Rho correlation coefficient = 0.021, *p* = 0.570).

### Concordance of PAT and PSG

**Fig. 2:**
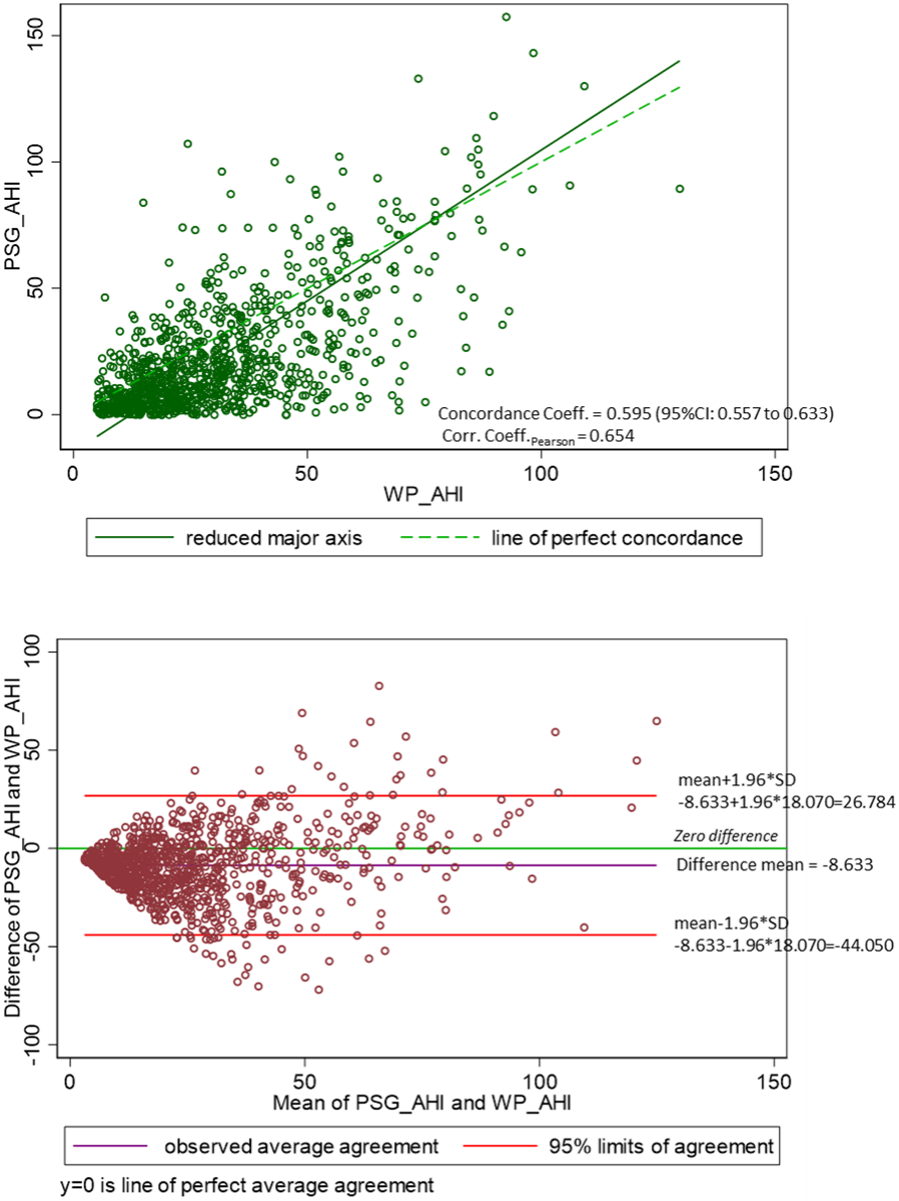
Concordance between AHI measured by PAT and AHI measured by PSG. **a** Scatter plot **b** Bland–Altman diagram. *PAT* peripheral arterial tonometry, *PSG* polysomnography

The concordance correlation coefficient for AHI measured by PAT and PSG was 0.595 (95% CI: 0.557 to 0.663) indicating a poor to moderate agreement between both sleep studies. The Pearson correlation between PAT and PSG AHI was 0.654 (Fig. 2a). In a Bland–Altman diagram, we showed that 6.0% (*n* = 56/940) of the data points fall outside the limits of agreement which were set at mean + 1.96 SD and − 1.96 SD. Further, it is shown that the PAT AHI measurements are in general higher (AHI + 8.6 ± 0.6/h) than that of PSG (Fig. 2b).

PAT had 80% concordance with PSG in the diagnosis of sleep apnoea. Of those patients with mild sleep apnoea in PAT 55% (*n* = 111/202) had sleep apnoea (of any severity) in the PSG, whereas when the PAT classified sleep apnoea as moderate or severe, the concordance with the PSG diagnosis of sleep apnoea was 86% (*n* = 637/738; Table 2).

**Fig. 3:**
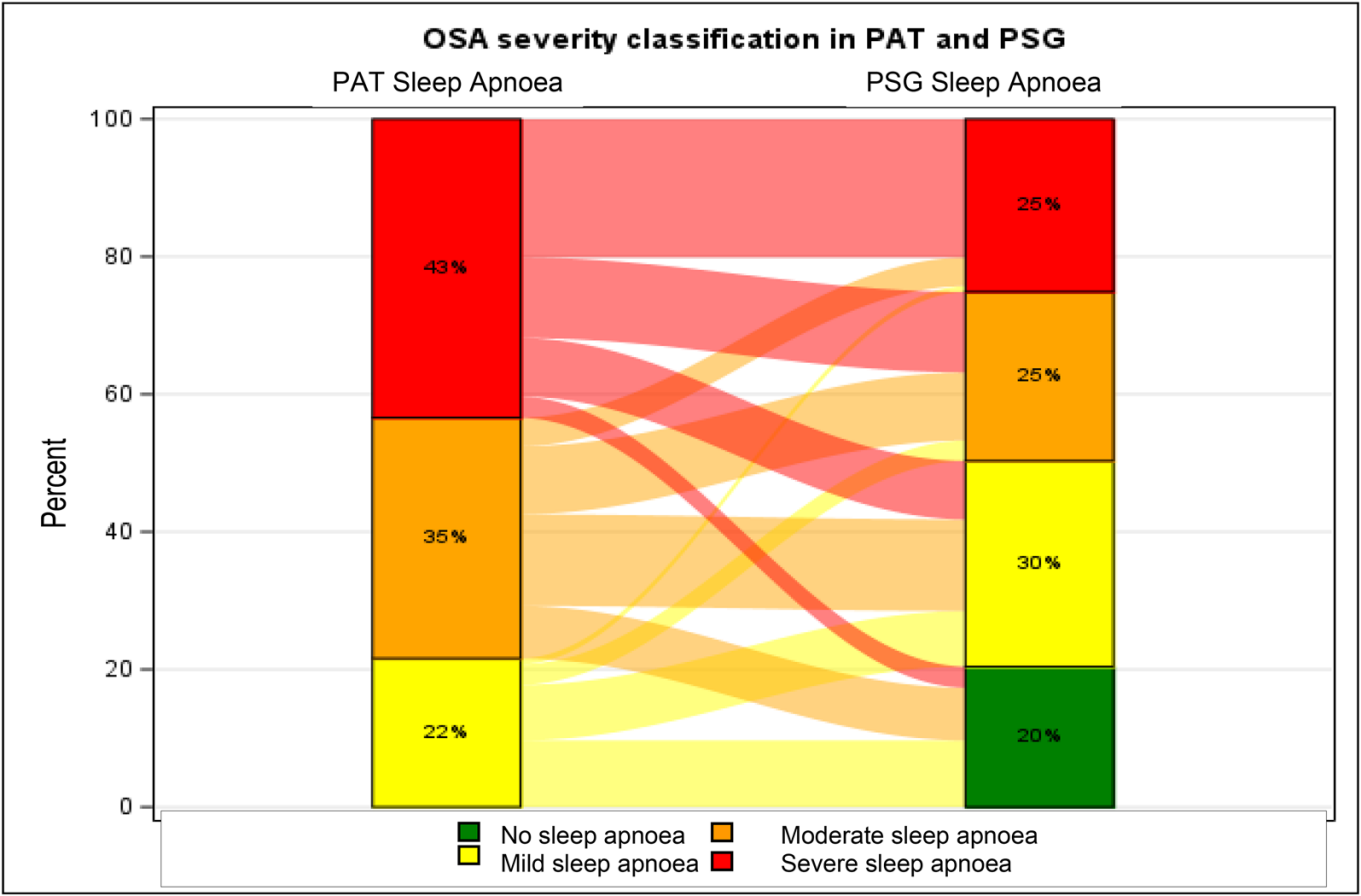
Obstructive sleep apnoea severity classification in PAT and PSG. *OSA* obstructive sleep apnoea, *PAT* peripheral arterial tonometry, *PSG* Polysomnography

In contrast to an overall good agreement in the diagnosis of sleep apnoea, our data showed differences in the classification of sleep apnoea severity between the two sleep studies. Based on PAT results, there was 37% (75/202) concordance for mild, 28% (93/330) for moderate, and 46% (189/408) for severe sleep apnoea **(**Fig. 3**)**.

Sleep variables assessed with the two sleep studies are shown in Table 3. PAT recorded longer total sleep time and less awake time than PSG. The percentage of snoring time was also significantly higher in PAT. On the other hand, the PSG recorded a longer time with oxygen saturation < 90%. Significant differences also exist for the sleep stages (light, deep, and REM sleep). It is important to note that the ODI in the PAT was automatically defined by an ODI of ≥ 4% whereas it is ≥ 3% in the PSG.

**Table 3:** Comparison of PAT and PSG sleep parameters stratified by matched concordant and discordant patients

Parameter	Concordant		*p*-value^a^	Discordant		*p*-value^a^
	PAT; avg ± SEM	PSG; avg ± SEM		PAT; avg ± SEM	PSG; avg ± SEM	
Sleep time (min)	345 ± 3.3	303 ± 3.0	**< 0.001**	351 ± 6.5	296 ± 6.1	**< 0.001**
AHI (/h)	34 ± 0.7	27 ± 0.9	**< 0.001**	19 ± 0.8	2 ± 0.1	**< 0.001**
ODI (/h)	22 ± 0.7	27 ± 1.1	**< 0.001**	9 ± 0.6	3 ± 0.3	**< 0.001**
Awake (%)	17 ± 0.3	26 ± 0.7	**< 0.001**	18 ± 0.7	24 ± 1.2	**< 0.001**
REM (%)	23 ± 0.3	16 ± 0.3	**< 0.001**	23 ± 0.6	16 ± 0.5	**< 0.001**
Deep sleep (%)	15 ± 0.3	21 ± 0.5	**< 0.001**	18 ± 0.5	25 ± 0.9	**< 0.001**
Time SpO_2_ < 90% (min)	28 ± 2.1	41 ± 2.6	**< 0.001**	15 ± 3.2	15 ± 2.9	0.158
Mean saturation (%)	93 ± 0.1	92 ± 0.2	**< 0.001**	94 ± 0.2	94 ± 0.2	0.123
Snore (%)	21 ± 0.9	10 ± 0.6	**< 0.001**	12 ± 1.5	3 ± 0.7	**< 0.001**

Values in bold indicate significant *p*-values

*AHI* apnoea hypopnoea index, *ODI* oxygen desaturation index, *REM* rapid eye movement, *PAT* peripheral arterial tonometry, *PSG* polysomnography, *SpO*_*2*_ oxygen saturation

^a^The Mann–Whitney *U*-test was used to evaluate the comparisons

### Comparison of cardiovascular outcomes between matched-concordant and matched-discordant patients

For 155 patients with discordant diagnosis for sleep apnoea between PAT and PSG (matched discordant), we found 274 controls (matched concordant) matched for age, sex, BMI and cardiovascular disease. There was a significant difference in baseline ESS score, coronary artery disease and liver disease between the groups (Additional file 1: Table S2). The differences in sleep parameters between matched concordant and matched discordant cases are shown in Additional file 1: Table S3.

The mean follow-up time was 3.1 ± 0.66 years. We detected 22 MACE, which is equivalent to an average of 1.5 MACE per 100 person years. There was no significant difference in the occurrence of MACE between matched concordant and matched discordant patients with or without prescribed PAP therapy (Table 4). Kaplan Meier analysis showed that matched concordant patients with a PAP device (*n* = 152) were protected against MACE and death (of any cause) compared to matched concordant cases without treatment (*n* = 122), *p* = 0.03.

**Table 4:** Follow-up parameters in patients with or without PAP therapy/prescription stratified by matched concordant and matched discordant measurements

	Matched concordant no PAP therapy *n* = 122; *n* (%), Mean ± SEM	Matched concordant with PAP therapy *n* = 152; *n* (%), Mean ± SEM	*p*-value^*^	Matched discordant no PAP therapy *n* = 144; *n* (%), Mean ± SEM	Matched discordant with PAP therapy *n* = 11; *n* (%), Mean ± SEM	*p*-value^*^
Follow-up time (days)	1184 ± 39	1184 ± 31	0.970	1190 ± 38	1167 ± 151	0.09
MACE after diagnosis	6 (5)	7 (5)	0.608	7 (4)	2 (18)	0.14
Death from any cause	4 (3)	2 (1)	0.412	2 (2)	1 (7)	0.20

*PAT* peripheral arterial tonometry, *PSG* polysomnography, *MACE* major adverse cardiovascular events

^*^p-values determined using the Chi-square test if variable is categorical and the Mann–Whitney *U*-test if continuous

### Clinical conditions associated with discordant OSA diagnosis between PAT and PSG

Factors significantly associated with a discordant or concordant diagnosis of sleep apnoea between PAT and PSG were age (*p* < 0.001), sex (*p* < 0.001), BMI (*p* < 0.001), arterial hypertension (*p* < 0.001), renal disease (*p* = 0.012), asthma (*p* = 0.044) and depression (*p* = 0.046). In a multivariate analysis adjusting for these seven parameters, only female sex (*p* < 0.001), younger age (*p* < 0.001) and lower BMI (*p* < 0.001) were statistically significant predictors of discordance.

In a linear, univariate analysis, arterial hypertension, asthma, chronic obstructive pulmonary disease (COPD) and congestive heart failure were significantly associated with AHI as assessed by PAT but not with AHI as assessed by PSG. Diabetes mellitus and atrial fibrillation were associated with both PAT AHI and PSG AHI (Table 5).

**Table 5:** Associations between clinical variables and AHI as measured by PSG and PAT

	PAT AHI		PSG AHI	
	Beta	*p*-value^a^	Beta	*p*-value^a^
Age (years)	0.164	**< 0.0001**	0.086	**0.008**
Body mass Index (kg/m^2^)	0.307	**< 0.0001**	0.275	**< 0.0001**
Pack years	0.149	**0.001**	0.108	**0.020**
Total sleep time (min)	− 0.126	**< 0.0001**	− 0.077	**0.018**
Time snoring (min)	0.251	**< 0.0001**	0.135	**0.001**
Comorbidities				
Arterial hypertension	0.101	**0.002**	0.061	0.062
Atrial fibrillation	0.074	**0.023**	0.070	**0.032**
Asthma	− 0.082	**0.012**	− 0.036	0.266
Chronic obstructive pulmonary disease	0.088	**0.007**	0.062	0.057
Congestive heart failure	0.100	**0.002**	0.021	0.512
Coronary artery disease	0.038	0.250	0.023	0.487
Depression	0.001	0.975	− 0.008	0.805
Diabetes mellitus	0.084	**0.010**	0.101	**0.002**

Values in bold indicate significant *p*-values

*AHI* apnoea hypopnoea index, *PAT* peripheral arterial tonometry, *PSG* polysomnography

^a^Linear regression

## Discussion

To date, this is the largest study comparing PAT with in-laboratory PSG in a representative clinical cohort of 940 patients. We show that PAT has a good concordance of 80% with PSG in diagnosing sleep apnoea. PAT AHI was on average higher than that of PSG (AHI + 8.6 ± 0.6/h). Several factors, including age, sex, and BMI, but not comorbidities, predicted diagnostic discordance between the two sleep studies. The MACE rate was similar in those with OSA diagnosed by PAT or PSG.

In most studies examining the accuracy of PAT, PSG was performed concurrently. Our sleep studies were performed on two different nights within a three-month period, which is in line with clinical practice when PSG is performed to objectify sleep apnoea diagnosis. This clinical approach explains why the correlation coefficient for AHI (PAT/PSG) in our study was 0.595 and markedly lower than reported in other studies [19, 31]. Nevertheless, concordance with PSG in diagnosing sleep apnoea was good and increased to 86% for diagnosis of sleep apnoea in patients with a PAT AHI score of ≥ 15/h. Limited accuracy of classifying sleep apnoea severity was recently reported [24, 31, 32]. This is also reflected in our data (Fig. 3). Focusing on therapeutic implications, data indicate that sleep apnoea of any stage should be treated if symptomatic [33]. In asymptomatic, non-sleepy OSA patients, it is less clear which subgroups benefit from treatment [34–37].

Relevant night-to-night variability of respiratory events must be considered if the sleep studies are not performed synchronously [38–40]. Positional differences in these two settings are important as it was reported that the supine position was overestimated in polysomnography compared to habitual sleep. This might influence sleep apnoea severity in patients with supine-predominant OSA [41]. Variability may also be caused by an intervening change in physical condition, such as increased leg fluid volume (rostral fluid shift) [42] and nasal obstruction [43]. Compared to an artificial in-laboratory assessment, HSAT in a familiar bed may positively influence the quality of sleep. Alcohol consumption may vary and affect sleep apnoea severity [44, 45]. For PAT performed in home or laboratory settings an AHI intraclass correlation coefficient of 0.75 (95% CI 0.62–0.84) was reported [46]. Factors that contributed to a divergence in diagnostic results from synchronous sleep testing included the presence of pulse oximetry artefacts and the misestimation of sleep time and arousal responses (decrease in PAT amplitude and increase in pulse rate) [23, 26, 31].

The pronounced differences in our study could also be partly related to a selection bias. We retrospectively analyzed real-life data reflecting medical decision making with patients. In our clinic, PAT was generally used as a screening device for patients with suspected OSA. Based on symptoms, comorbidities, patient preferences and PAT results, further diagnostic testing with PSG was discussed. OSA treatment was rarely initiated without confirmatory PSG. It can be assumed that there is a selection for more symptomatic patients with comorbidities and abnormal PAT findings.

Patient characteristics that were significantly associated with discordance in the diagnosis of sleep apnoea between PAT and PSG included sex (female), younger age and lower BMI. The influence of comorbidities on the validity of the PAT assessment is still controversial. While Kinoshita et al. demonstrated a limited AHI correlation with increased arterial stiffness [25], Zhang et al. described a lower PAT-PSG concordance in the elderly (≥ 65 years) and discussed their results in the context of comorbidities and vascular compliance [26]. Studies focusing on patients with concomitant diseases such as atrial fibrillation or COPD nevertheless described good (PAT/PSG) AHI correlations [20, 47]. The study of Ioachimescu et al. in a clinical cohort found no significant influence of concomitant diseases (such as asthma, COPD, congestive heart failure and atrial fibrillation) on the performance of PAT-based testing [31]. In our study, comorbidities such as arterial hypertension, congestive heart failure, atrial fibrillation, COPD and diabetes mellitus did not predict divergent results nor were they associated with bigger differences in AHI between the two diagnostic methods. When adjusted for age, BMI and sex this also applied to asthma and depression. It is important to note that the PAT version used in our study could not distinguish between central and obstructive sleep apnoea. Although the overall AHI should not be affected, an elevated AHI may also reflect central breathing events, especially in the presence of concomitant diseases such as heart failure [48, 49].

Discordance in sleep apnoea diagnosis was particularly high in mild sleep apnoea around the AHI cut-off value of 5/h, so it is not surprising that younger age and lower BMI were associated with diagnostic divergence. Based on several population prevalence studies women (especially premenopausal women) tend to have a lower mean prevalence of OSA than men [50, 51].

The AASM recommends HSAT as a diagnostic test for OSA in uncomplicated patients with an increased pre-test probability [17]. Increased risk of (moderate to severe) OSA was defined by daytime sleepiness in combination with two additional criteria such as loud snoring, apnoea or choking and arterial hypertension [17]. Uncomplicated patients were defined by the absence of significant cardiopulmonary disease, respiratory muscle weakness (neuromuscular condition), history of stroke, opioid medication, risk of (sleep-related) hypoventilation, etc. [17].

In our clinical cohort, PAT led to a diagnosis of sleep apnoea in 940 patients, 20% of whom had a negative PSG and were classified as discordant. Performing a follow-up we analysed whether these discordant patients showed a different cardiovascular risk. Our data indicate a similar MACE risk in matched-discordant (PAT positive, PSG negative) and matched-concordant (PAT positive, PSG positive) patients with or without PAP therapy. In the context of this study, we could not distinguish whether the impact of OSA (PSG AHI > 5/h) was too small to cause a significant difference in cardiovascular outcomes [34, 35] or whether these matched-discordant patients were a group at increased cardiovascular risk, similar to those with OSA diagnosed with PSG.

The present study has some limitations. Since PAT was used to screen for OSA, we excluded patients with a PAT AHI < 5/h for further analysis. Therefore, we cannot make any reliable statement about the sensitivity or specificity. In 30 patients with a negative PAT a PSG was performed within 3 months due to increased clinical suspicion of sleep apnoea (data were neither shown nor integrated into the analysis). In this subgroup 8 (27%) had mild and 3 (10%) had moderate OSA. No severe sleep apnoea could be objectified in this subgroup. Comparable data for negative PAT were found in the PATER study [31]. Previous studies have shown that PAT has a good sensitivity for an AHI ≥ 5/h (pooled sensitivity of 96%; CI 93–97%) [52]. The focus of our study was to determine how a positive PAT result (≥ 5 /h) in a representative clinical cohort of different pre-test probabilities, could be integrated into medical decision-making and showed a good PPV of 0.8 for a PAT AHI ≥ 5/h or 0.86 for an AHI ≥ 15/h. Following the AASM recommendations [17], our data support the approach that HSAT with PAT can be used in daily clinical practice to diagnose obstructive sleep apnoea in patients with signs and symptoms of OSA in the absence of risk factors for other sleep-related breathing disorders when the AHI in PAT is ≥ 15/h.

In our mean follow-up period of 3.1 ± 0.06 years, MACE occurred at an average of 1.5 events per 100 person years, which is lower than in other studies of sleep apnoea patients in which 2.1–4.5 events per 100 person years were observed [34, 53]. Considering that our tertiary centre is the largest medical centre in the region, the primary access point for patients with acute coronary syndrome and stroke, and that the primary care physicians were contacted to supplement in-house data, we assume that the majority of cardiovascular events for our subpopulation were captured. The difference in our results compared to published values could be due to differences in comorbidities, age of the population and differences in the definition of what constitutes a MACE. Considering our data with similar cardiovascular risks, similar ESS score in both groups (matched concordant ESS 9.3 ± 0.3 and discordant ESS 9.2 ± 0.4), and the fact that PSG outcomes are also influenced by several factors such as night-to-night variability, it would be an interesting question to conduct a randomized treatment trial in symptomatic PAT-positive PSG-negative patients.

## Conclusions

Home sleep apnoea testing with PAT is increasingly used in clinical studies and practice for the diagnosis of OSA. In a representative, heterogeneous and large clinical population we found a good concordance of 80% in sleep apnoea diagnosis with a consecutive PSG, which increased to 86% with a PAT AHI of ≥ 15/h. In a retrospective follow-up of MACE, PAT positive (AHI ≥ 5/h) and PSG negative (AHI < 5/h) patients showed a similar cardiovascular risk as patients with PSG-confirmed OSA.

## Supplementary Information

**Additional file 1:** **Table S1.** Sleep apnoea syndrome calculated using AHI ≥5 or 15 and ESS ≥10. **Table S2.** Descriptive statistics for patients characteristics stratified by matched discordant and concordant cases. **Table S3.** Comparison of sleep parameters between matched concordant and discordant patients stratified by measurement method used.

## Data Availability

The datasets used and/or analysed during the current study are available from the corresponding author on reasonable request.

## Abbreviations

AHI: Apnoea-hypopnoea index
AASM: American Academy of Sleep Medicine
BMI: Body mass index
COPD: Chronic obstructive pulmonary disease
HSAT: Home sleep apnoea testing
MACE: Major adverse cardiovascular events
ODI: Oxygen desaturation index
OSA: Obstructive sleep apnoea
OSAS: Obstructive sleep apnoea syndrome
PAP: Positive airway pressure
PAT: Peripheral arterial tonometry
PSG: Polysomnography
RDI: Respiratory disturbance index
