# Supplementary material for: Peripheral arterial tonometry versus polysomnography in suspected obstructive sleep apnoea
Source: Eur J Med Res. 2023 Jul 22;28:251. doi: 10.1186/s40001-023-01164-w (PMC10362713; doi:10.1186/s40001-023-01164-w)
Supplement: Supplementary file 1 — Additional file 1: Table S1. Sleep apnoea syndrome calculated using AHI ≥5 or 15 and ESS ≥10. Table S2. Descriptive statistics for patients characteristics stratified by matched discordant and concordant cases. Table S3. Comparison of sleep parameters between matched concordant and discordant patients stratified by measurement method used. [file 40001_2023_1164_MOESM1_ESM.docx]

**Peripheral arterial tonometry versus polysomnography in suspected obstructive sleep apnoea**

Online Data Additional file

**Table S1: Sleep apnoea syndrome calculated using AHI ≥5 or 15 and ESS ≥10**

|  |  | **PSG sleep apnoea syndrome using AHI≥5 and ESS≥10** | |  |
| --- | --- | --- | --- | --- |
|  |  | No | Yes | Total |
| **PAT sleep apnoea syndrome using AHI ≥5 and ESS ≥10** | No | 392 (52) | 0* | 754 |
|  | Yes | 71 (9) | 291 (39) |  |
|  |  | **PSG sleep apnoea syndrome using AHI ≥15 and ESS ≥10** | |  |
|  |  | No | Yes | Total |
| **PAT sleep apnoea syndrome using AHI ≥15 and ESS ≥10** | No | 315 (54) | 0* | 584 |
|  | Yes | 105 (18) | 164 (28) |  |

*Due to the exclusion of negative PATs, a bias is present

AHI= apnoea hypopnoea index, ESS= Epworth sleepiness scale, PAT= Peripheral arterial tonometry, PSG= Polysomnography

**Table S2: Descriptive statistics for patients characteristics stratified by matched discordant and concordant cases**

|  | Matched concordant  All (n=274); n(%) | Matched discordant  All (n=155); n(%) | p-value* |
| --- | --- | --- | --- |
| Age (years) | 52.4±0.8 | 52.2±1.1 | 0.171 |
| Sex: Male | 179 (65) | 99 (64) | 0.488 |
| Body Mass Index (kg/m^2^) | 30±0.4 | 29.2±0.48 | 0.368 |
| Epworth Sleepiness Score | 9.3±0.3 | 9.2±0.4 | **0.012** |
| Pack years | 24.1±1.7 | 25.7±2.4 | 0.621 |
| Smoking status | n=251 | n=138 | 0.191 |
| Current / ex-smoker | 130 (52) | 81 (59) |  |
| Never smoker | 121 (48) | 57 (41) |  |
| Comorbidities |  |  |  |
| Arterial hypertension | 140 (51) | 74 (48) | 0.505 |
| Asthma bronchiale | 32 (12) | 28 (18) | 0.067 |
| Atrial fibrillation | 10 (4) | 7 (5) | 0.906 |
| Cerebral vascular disease | 24 (9) | 9 (6) | 0.270 |
| Chronic obstructive pulmonary disease | 19 (7) | 19 (12) | 0.062 |
| Congestive heart failure | 11 (4) | 11 (7) | 0.164 |
| Coronary artery disease | 22 (8) | 22 (14) | **0.043** |
| Depression | 37 (14) | 32 (21) | 0.053 |
| Diabetes mellitus | 42 (15) | 21 (14) | 0.617 |
| Liver disease | 11 (4) | 14 (9) | **0.033** |
| Renal disease | 32 (12) | 14 (9) | 0.397 |
| Rheumatological disease | 18 (7) | 11 (7) | 0.834 |

*Differences determined using the Chi-squared test if variable is categorical and the Mann-Whitney U-test if continuous

**Table S3: Comparison of sleep parameters between matched concordant and discordant patients stratified by measurement method used**

|  | **PAT** | | **p-value*** | **PSG** | | **p-value*** |
| --- | --- | --- | --- | --- | --- | --- |
|  | **Matched concordant n=274; avg±SEM** | **Matched discordant n=155; avg±SEM** |  | **Matched concordant n=274; avg±SEM** | **Matched discordant n=155; avg±SEM** |  |
| **Sleep time (min)** | 345 ± 4.6 | 347 ± 7.1 | 0.353 | 316 ± 4.6 | 289 ± 7.2 | **0.023** |
| **AHI (/h)** | 32 ± 1.2 | 19 ± 1.0 | **<0.001** | 25 ± 1.3 | 2 ± 0.1 | **<0.001** |
| **ODI (/h)** | 21 ± 1.1 | 9 ± 0.6 | **<0.001** | 24 ± 1.4 | 3 ± 0.3 | **<0.001** |
| **Awake (%)** | 16 ± 0.5 | 19 ± 0.8 | **0.020** | 22 ± 0.9 | 26 ± 1.4 | 0.234 |
| **REM (%)** | 23 ± 0.5 | 23 ± 0.7 | 0.861 | 16 ± 0.5 | 16 ± 0.6 | 0.804 |
| **Deep sleep (%)** | 16 ± 0.5 | 18 ± 0.5 | **0.002** | 21 ± 0.7 | 25 ± 1.0 | **<0.001** |
| **Time SpO_2_ < 90% (min)** | 24 ± 3.5 | 13 ± 3.2 | **<0.001** | 31 ± 4.0 | 18 ± 3.6 | **<0.001** |
| **Mean saturation** | 93 ± 0.1 | 94 ± 0.2 | **0.019** | 93 ± 0.2 | 93 ± 0.2 | **<0.001** |
| **Snore (%)** | 21 ± 1.5 | 13 ± 1.7 | **<0.001** | 11 ± 1.2 | 5.8 ± 1.4 | **<0.001** |

*Mann-Whitney U-test used to determine differences

AHI= apnoea hypopnoea index, ODI= oxygen desaturation index, REM= rapid eye movement, PAT= Peripheral arterial tonometry, PSG= Polysomnography, SpO_2_= oxygen saturation
